# Fracture and Fatigue of Titanium Narrow Dental Implants: New Trends in Order to Improve the Mechanical Response

**DOI:** 10.3390/ma12223728

**Published:** 2019-11-12

**Authors:** Eugenio Velasco-Ortega, Antonio Flichy-Fernández, Miquel Punset, Alvaro Jiménez-Guerra, José María Manero, Javier Gil

**Affiliations:** 1 Department Estomatologia, Facultad de Odontología, Universidad de Sevilla, 41009 Sevilla, Spain; evelasco@us.es (E.V.-O.); alopajanosas@hotmsil.com (A.J.-G.); 2 Departament of Estomatología, Facultad de Medicina y Odontología, Universidad de Valencia, 46010 Valencia, Spain; aflichy@uv.es; 3 Department of Materials Science and Metallurgical Engineering, Universitat Politècnica de Catalunya (UPC), 08019 Barcelona, Spain; miquel.punset@upc.edu (M.P.); jose.maria.manero@upc.edu (J.M.M.); 4 UPC Innovation and Technology Center (CIT-UPC), 08028 Barcelona, Spain; 5 Bioengineering Institute of Technology, Universitat Internacional de Catalunya, 08195 Barcelona, Spain

**Keywords:** narrow dental implants, titanium, fatigue, fracture, plastic deformation

## Abstract

Sixty-four fractured commercially pure titanium (cp-Ti) narrow dental implants (NDIs) with similar macrogeometry and connection designs were studied after different implantation times in humans in order to determine their reliability and to evaluate the causes of the fracture. These NDIs were compared with other similar implants, made with alloyed titanium with 15% Zr and with 12% strained titanium. Original implants were tested under static and fatigue conditions, simulating the tri-axial loads in the mouth by means of a Bionix hydraulic test machine. Fractography was studied using field-emission scanning electron microscopy (FSEM). The results showed that cp-Ti NDI exhibits low strength for mechanical cycling, and the alloyed Ti and strained titanium increase the mechanical strength, guaranteeing long term mechanical behavior. NDIs fractured due to fatigue, and, in some cases, the presence of cracks in the original NDIs quickly led to fracture. These cracks were attributed to plastic deformation during machining were found to be exacerbated due to acid etching in the passivation process. All cases of fracture were cp-Ti dental implants due to the low fatigue limit. The results show that, when titanium is alloyed or cold-worked, the fatigue limit is higher than cp-Ti. This in vitro research will help clinicians to select a better NDI system for safer treatment.

## 1. Introduction

Sometimes, the available bone tissue is insufficient to place regular-diameter dental implants, and new surgical techniques are necessary for increasing and regenerating the hard tissue. The use of narrow dental implants (NDIs) is an alternative treatment, because these allow dental restorations in areas with limited prosthetic space and they can be inserted in places that would otherwise require grafting techniques [1,2,3,4]. In recent studies, the fatigue failure of narrow implants with different implant–abutment connection designs was analyzed. From the results of this study, it can be observed that differences occur between external and internal connections, with external connections having the lowest reliability. The fatigue results with different internal connections were not significantly different between groups [5]. Hirata et al. [6] evaluated the survival probability of four narrow-diameter implant systems when subjected to fatigue loading. All implants were made with commercially pure titanium (cp-Ti) grade 4 with similar designs and diameters from 3.3 to 3.5 mm, with different geometries of internal connections and abutments. The survival probability was not significantly different among the implant systems. 

**Figure 1:**
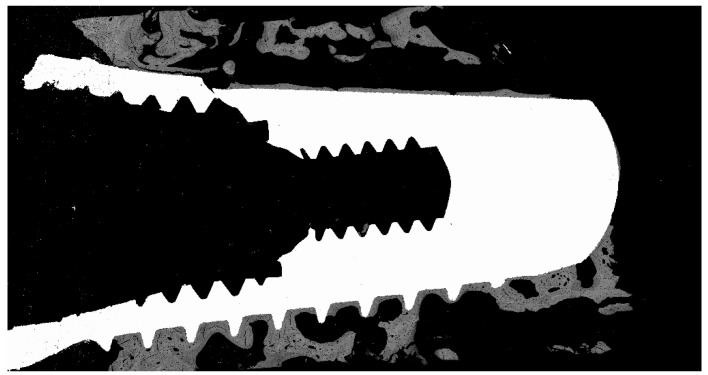
SEM micrographs of a narrow dental implant (NDI) fractured 17 months after implantation in a patient. The fracture is located in the connection zone where the cross-sectional surface is lower.

The mechanical strength of cp-Ti is limited and its use in NDIs increases the risk of fracture of the Ti dental implant [7,8,9]. In Figure 1, the fracture of an osseointegrated NDI after 17 months of implantation in a patient can be observed. The small diameter and reduced wall thickness, and the lower material bulk hamper the long-term survival. Furthermore, the bending of the prosthetic components increases, thereby decreasing the restoration reliability.

It is well known that external hexagon connections are not adequate due to loads with different angles drastically reducing the strength. Internal conical or hexagonal connections offer an improvement; however, abutment neck fractures can even be observed in screwed internal connections, with some involving implant fractures [3,7,10,11,12,13,14,15,16].

The objective of this research was firstly to study the cause of such premature fractures of NDIs made from cp-Ti grade 4. Secondly, an evaluation of the mechanical properties, with suggestions for their improvement, was carried out following two strategies: (1) alloying the titanium with 15% Zr, and (2) straining the cp-Ti at 12%. Both strategies were employed to increase the mechanical strength, hardness, and the fatigue life, so as to obtain a stress value at which the sample supported a total of 10^7^ cycles, which was considered the fatigue limit.

## 2. Experimental Results and Discussion

The roughness (Ra) was very similar for the different dental implants studied: ~1.6 µm for cp-Ti implants, ~1.7 µm for alloyed implants, and ~1.9 µm for cold-worked implants. The mechanical properties of the tested implants are shown in Table 1. The yield strength for the grade 4 cp-Ti was lowest, and it increased for the other implants. A similar trend was observed for the maximum strength (σ_max_). On the other hand, the strain to fracture was lower for the Ti alloy and cold-worked Ti. Ti-15%Zr presented a significantly higher value of strain compared to the cold-worked Ti [13,14,15,16,17,18,19,20]. Consequently, the cold-worked Ti presented a lower value of toughness, without any effects on other properties such as the biocompatibility or corrosion resistance of cp-Ti. The hardness was shown to be significantly higher for the cold-worked Ti than the other implants, due to the increase in the number of linear defects in the hexagonal microstructure of α-titanium. The density of slip dislocations produced an increase in the hardness and the mechanical properties. Ti-15%Zr also presented significantly higher hardness values than cp-Ti grade 4 [21,22,23,24].

**Figure 2:**
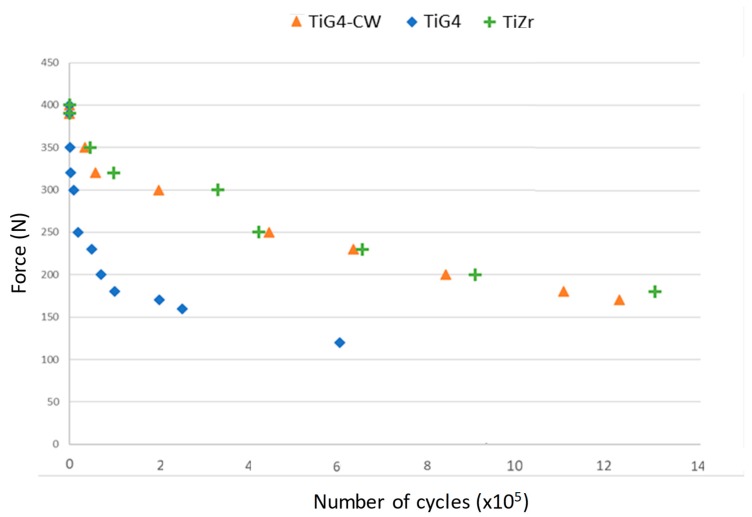
S–N curves (stress vs. number of cycles) of the different NDIs studied.

In fatigue tests, material behavior is commonly characterized by an S–N curve, which describes the magnitude of a cyclic force or stress (S) as a function of the number of cycles to failure (N). A value under the curve suggests that a dental implant will not fracture. Figure 2 shows the S–N curve for the different NDIs studied. The results show that the implants alloyed with Zr and those with Ti grade 4 submitted to 12% cold working presented more fatigue life than the Ti dental implants (grade 4) without hardening treatment. The cold-worked or alloyed implants presented an asymptotic curve around 200 N. However, the cp-Ti grade 4 presented a curve around 100 N. These values demonstrate the importance of the static mechanical properties on the cyclic behavior. This aspect is very important in clinical applications, whereby cp-Ti for NDIs would produce premature fractures when chewing loads exceed 100 N.

**Figure 3:**
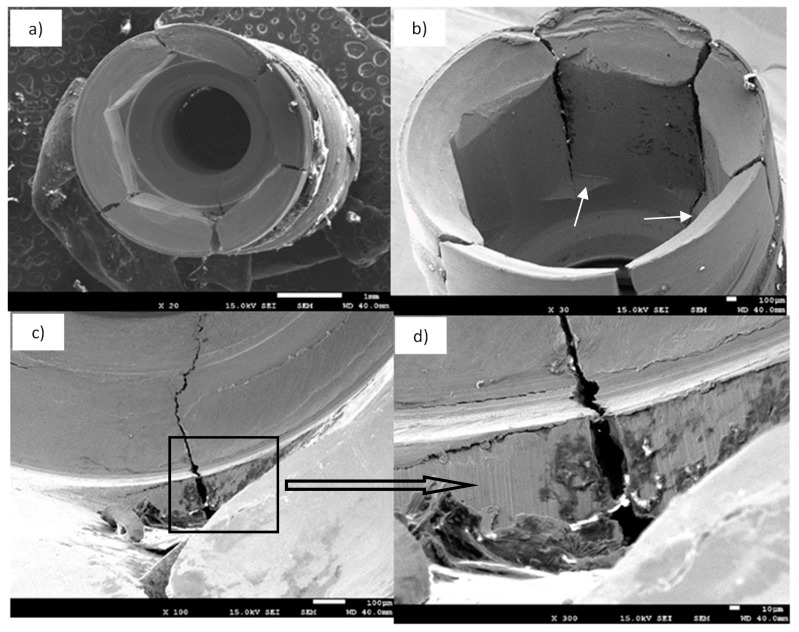
SEM fractography of explanted cp-Ti grade 4 NDIs: (**a**) SEM micrograph of an explanted implant, top view; (**b**) SEM micrograph of hexagonal inner connection (side view) showing longitudinal fracture cracks; the white arrows mark the places of crack nucleation; (**c**,**d**) SEM micrographs showing crack propagation at different magnifications.

For NDIs which fractured after being inserted in patients, it can be observed that the fracture mechanism was fatigue in all cases. The fractography can be observed in Figure 3, with different SEM micrographs showing a detailed description of both crack nucleation points and crack propagation paths. Figure 3 shows the presence of many longitudinal cracks along practically all vertices between the walls of the inner hexagonal connection [25,26,27]. 

The crack nucleation was generated inside the implant body, specifically in the inner hexagonal connection walls just inside the bottom horizontal ground plane. After crack nucleation, progressive crack growth produced under cyclic masticatory multiaxial loading was responsible for the propagation path along the vertices of hexagonal connections, as well as through the walls, as can be seen in Figure 3b [28,29,30].

Further fractography analysis performed on fatigue-tested NDIs showed the same fracture patterns, as well as the same crack nucleation points and fracture paths. 

**Figure 4:**
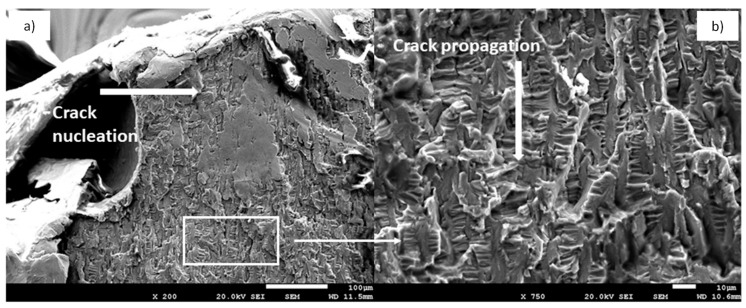
SEM micrographs of the cpTi grade 4 NDI showing crack nucleation and propagation: (**a**) SEM micrograph showing crack nucleation starting point, (**b**) SEM micrograph showing crack cyclic propagation due to fatigue.

From Figure 4a, the site of crack nucleation can be observed, as well as a site where crushing occurred, due to the friction between the fracture surfaces in the crack propagation. This was the least effective surface to which the load was applied and, consequently, this was the point of maximum stress which provoked crack nucleation in the load cycle. The subsequent crack propagation was fast due to the cyclic loads, leading to fracture. In Figure 4b, the typical striation or fatigue marks indicating the direction of crack propagation can be observed [15,16,17,18,29].

**Figure 5:**
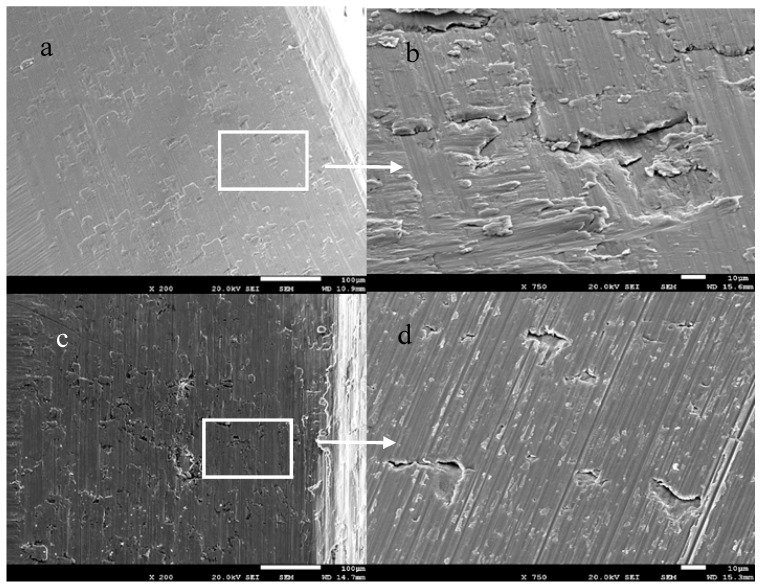
SEM micrographs of inner cp-Ti grade 4 implant connection, showing some plastic deformation and crack formation on the connection surface due to “broaching”. (**a**). Defects in the internal surface. (**b**). Defects with more detail from 5a. These defects are the place of the crack nucleation. (**c**). Defects in the internal surface with more plastic deformation. (**d**). These defects are cracks.

One cause of premature fracture in the cp-Ti NDIs was the poor mechanical properties, especially in the connection zone, in relation to the other implants, which had higher strength and fatigue limits. On the other hand, in the manufacturing process, the conformation of hexagonal connections results from plastic strain. This plastic strain produces small defects, as shown in Figure 5. These defects present a higher residual energy and are the points of crack nucleation. Furthermore, the implants are treated with acids in order to increase their roughness, so as to improve osseointegration and passivate the dental implant. Sometimes, acid etching is used to color the connection, thereby helping the surgeon identify the pieces for prosthesis. These acids, therefore, etch the defects and produce cracks before the insertion of the implant into the patient. When the implant is inserted, crack propagation starts due to chewing cyclic loads until fracture occurs [19,20,21,30,31,32].

## 3. Materials and Methods

**Figure 6:**
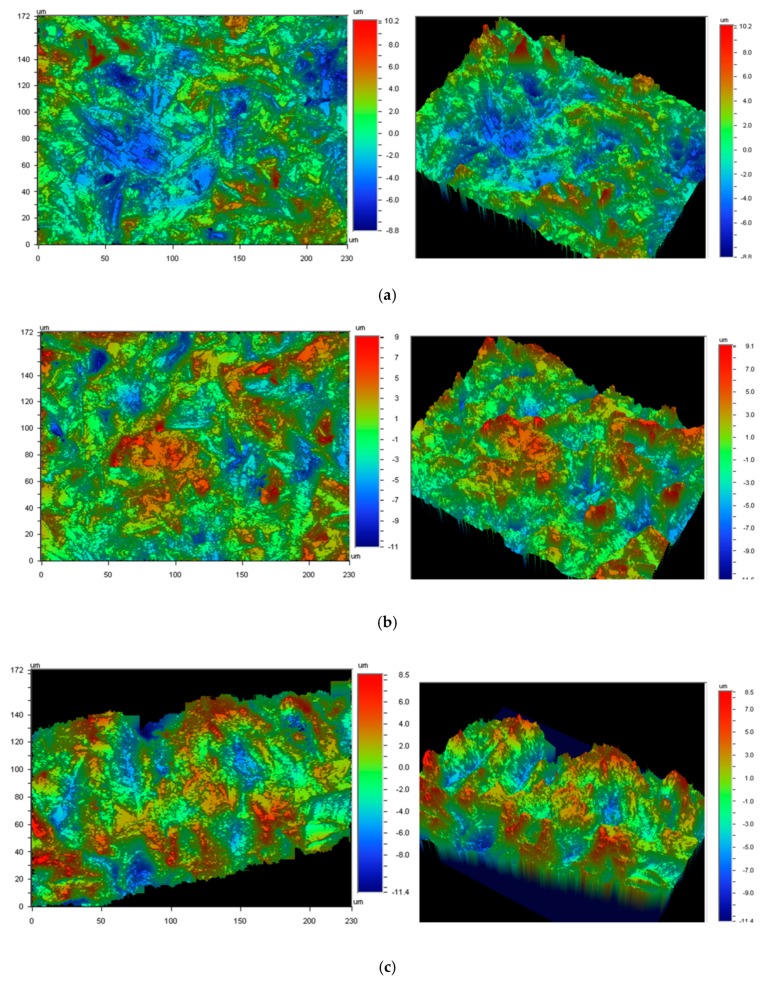
Roughness of the different surfaces studied. (**a**) cp-Ti grade 4 dental implant; (**b**) Ti-15%Zr dental implant; (**c**) cold-worked (12%) dental implant. These topographies were obtained by sandblasting with Al_2_O_3_ and acid etching.

Sixty-four cp-Ti grade 4 fractured NDIs were studied. All subjects gave their informed consent for inclusion before they participated in the study. The study was conducted in accordance with the Declaration of Helsinki, and the protocol was approved by the Ethics Committee with the code CUO-R-2019-2310. The dental implants had screwed internal connections. The surface was treated by sandblasting, using Al_2_O_3_ particles as an abrasive material, as well as by acid-etching, in order to achieve roughness (Ra). In Figure 6, the original NDI during fabrication can be observed. Roughness was evaluated for the test surfaces using white-light interferometer microscopy (Wyko NT1100, Veeco, Tucson, AZ, USA). The analyzed surface area was 459.9 × 604.4 µm^2^ for all microrough surfaces. Data analysis was performed with Wyko Vision 232TM software (version 4.0, Veeco, Tucson, AZ, USA). A Gaussian filter was used to separate waviness from the roughness of the surface. Cut-off values, λc = 0.8 mm, for microrough surfaces were applied. The measurements were made on three different surfaces for each type of surface treatment to characterize the Ra (the average roughness), which was calculated as the arithmetic average of the absolute values of the distance of all points of the profile to the mean line. 

**Figure 7:**
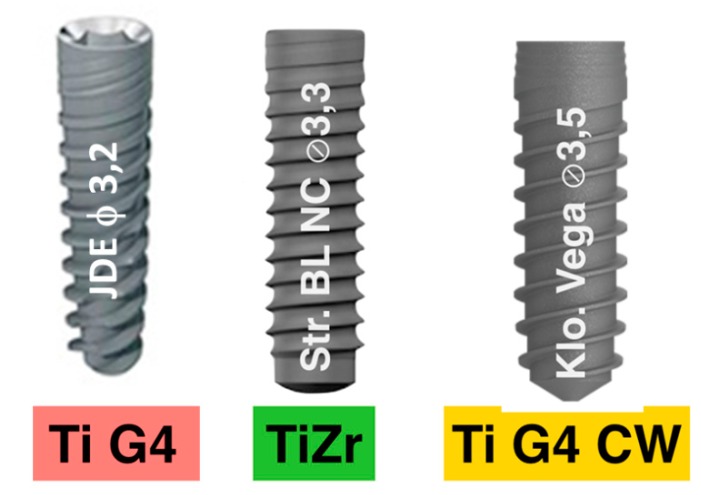
NDIs studied. TiG4: cp titanium grade 4; TiZr: titanium alloyed with 15% Zr; TiG4CW: cp titanium grade 4 cold-worked at 12%.

For comparison with other NDIs which were submited to a hardening processes, such as alloying with 15% Zr or cold-working at 12%, Roxolid^®^ by Straumann (Switzerland) and Vega^®^ by Klockner (Spain) were used, respectively. The designs and main characteristics are presented in Figure 7 and Table 2. As can be observed, the macrodesign and roughness were very similar. 

Initially, static uniaxial compression tension tests were conducted in order to determine the yield strength of the material, the ultimate strength, and the strain to fracture. The hardness of the specimens was measured on polished cross-sections using a Vickers microhardness tester (Akashi, Matsusawa, Japan) with a Vickers diamond indenter under a load of 0.98 N (100 gf) and 15 s of indentation. Fifteen data points were collected and averaged for each hardness value. Ten implants were analyzed for static tension, and the same number was used for hardness testing (*n* = 10).

Samples used in hardness testing were firstly embedded in methyl-methacrylate resin (Technovit 7200; Kulzer-Heraus GmbH, Wehrheim, Germany), and subsequently photo-polymerized in a light control unit (Histolux; Kulzer GmbH, Wehrheim, Germany) in order to obtain solid transparent blocks to allow sample cutting and polishing, thereby avoiding any kind of deformation and/or fracture during these procedures. Resin blocks were cut along their cross-section using a diamond saw EXAKT 310 CL (EXAKT Advanced Technologies GmbH, Norderstedt, Germany) with continuous water irrigation at a maximum rotation speed with minimum load. Finally, cross-section samples were polished by means of an automatic grinding machine Exakt-400CS (EXAKT Advanced Technologies GmbH, Norderstedt, Germany), with parallelism, load, and speed controls, firstly using SiC papers increasing in abrasiveness (600, 800, and 1200 grit), and subsequently polishing using a 1 µm Al_2_O_3_ abrasive suspension, following the recommendations of the ASTM-E3 standard [22].

**Figure 8:**
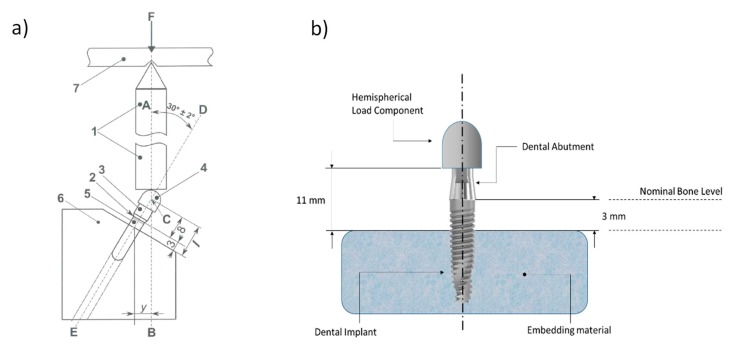
(**a**) Overall scheme of the mechanical testing described by the ISO 14801 standard. (**b**) Front view of representative drawing with lengths and distances of the testing samples.

Implants used for mechanical testing were embedded into a polymeric resin to mimic oral conditions before performing any mechanical assay in order to provide a stable support. NDIs were placed perpendicularly at 3.0 mm ± 0.1 mm above the nominal bone level described by the different manufacturers in a cold auto-curing polymeric resin (Mecaprex MA2+, Presi, France), as shown in Figure 8.

All NDI systems used for mechanical testing were assembled according to different surgical protocols, using the correct original components (abutments, screws, and implants), as well as using proper torques defined by each manufacturer with self-adjustable precision surgical tools.

Following the static compression-to-fracture tests, fatigue tests at various percentages of the obtained yield strength were performed following the recommendations of the ISO 14801:2016 standard [23], which allowed determining the number of cycles before fracture. The assays were performed with a servo-hydraulic testing machine (MTS Bionix 858, Minneapolis, MN, USA) equipped with the software TestStar II (MTS, Minneapolis, MN, USA). This machine was equipped with a load cell (MTS) of 25 kN. The implants were loaded with a sinusoidal function of fatigue at a frequency of 15 Hz and 10% stress variation. 

**Figure 9:**
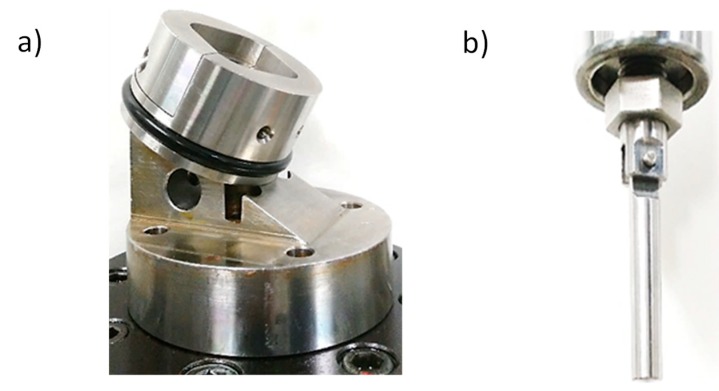
Detail of the fatigue testing grips used by servo-hydraulic testing machine: (**a**) lower clamping grip; (**b**) upper articulated grip.

The dynamic cyclic fatigue assay was carried out in a triaxial compression–flexion–torsion mode, placing the NDI implants fixed with an inclination of 30° ± 2° of the compression stress application axis with the *z*-axis with respect to the tensile compression machine (Figure 9), under sinusoidal load at 30° and 15 Hz at room temperature under dry conditions [33]. The data were represented as the number of cycles reached at fracture for different applied forces. The deformed and fractured specimens were observed by means of field-emission scanning electron microcopy (JSM 7100, Jeol, Tokyo, Japan).

Statistically significant differences among test groups for mechanical evaluation were assessed using statistical software (MinitabTM 13.1, Minitab Inc., New York, NY USA). ANOVA tables with a multiple comparison Fisher test were calculated. The level of significance was established at a *p*-value <0.05.

All 64 fractured implants remained in position for a period of time ranging from six weeks to 20 months, after which the dental implants were fractured in all cases in the connection. The patients were between 35 and 74 years old (58% women and 42% men). All patients were treated with calcium phosphate granules in order to regenerate the bone. Once bone formation was obtained, a new dental implant was implanted.

## 4. Conclusions

All cases of fractured narrow dental implants were manufactured with cp-Ti, and the failure mechanism was fatigue in all cases. The mechanical properties of cp-Ti NDIs should be improved in order to guarantee long-term success of the treatment. Different premature fractures were observed by fatigue due to the poor mechanical properties and the defects in the surfaces produced during the conformation process. The hardening methods studied (cp-Ti with 12% cold-work straining or alloying with 15% Zr) showed higher fatigue limits than cp-Ti, thereby ensuring good mechanical reliability. Cp-Ti should be used with caution by clinicians, and this study will help clinicians to select a better NDI system for a more predictable treatment.

## Figures and Tables

**Table 1:** Mechanical properties of the dental implants studied, being σ_max_: Maximum strength (MPa), σ_0.2_: Yield stress (MPa), ε: Strain to fracture (%), HVN: Hardness Vickers number.

	Implant	σ_max_ (MPa)	σ_0.2_ (MPa)	ε (%)	HVN
cp-Ti (grade4)	JDE	460 (37)	357 (23)	17 (4)	104 (12)
Ti-15%Zr	Roxolid	877 (24)	678 (20)	22 (4)	199 (15)
Ti strained 12%	Vega	1100 (35)	740 (23)	8 (2)	380 (23)

**Table 2:** Implants used in the experiment.

Group	Group 1 Cp-Ti Grade 4	Group 2 Ti Alloyed with 15%Zr	Group 3 Cp-Ti Grade 4 Hardened by 12% Cold-Working
Implant type	JDE (Ø 3.2 mm, h 8 mm) (JDental, Modena, Italy)	Bone level Roxolid (Ø 3.3 mm, h 8 mm) (Straumann AGR, Basel, Switzerland)	Bone level Vega (Ø 3.5 mm, h 8mm) (Klockner, Spain)
Connection type	Conical internal	Cross-fit internal	Hexagon internal

## Abbreviations

FSEM	Field-emission scanning electron microscope
HVN	Hardness Vickers number
NDI	Narrow dental implants
Ra	Average surface roughness (µm)
SEM	Scanning electron microscope
cp-Ti	Commercially pure titanium
σ_0.2_	Yield stress (MPa)
σ_max_	Maximum strength (MPa)
ε	Strain to fracture (%)
